# MT-Feeding-Induced Impermanent Sex Reversal in the Orange-Spotted Grouper during Sex Differentiation

**DOI:** 10.3390/ijms19092828

**Published:** 2018-09-19

**Authors:** Qing Wang, Minwei Huang, Cheng Peng, Xiang Wang, Ling Xiao, Dengdong Wang, Jiaxing Chen, Huihong Zhao, Haifa Zhang, Shuisheng Li, Huirong Yang, Yun Liu, Haoran Lin, Yong Zhang

**Affiliations:** 1 College of Marine Sciences, South China Agricultural University, Guangzhou 510642, China; wangqing@scau.edu.cn (Q.W.); zhaohh@scau.edu.cn (H.Z.); 2 State Key Laboratory of Biocontrol, Guangdong Provincial Key Laboratory for Aquatic Economic Animals and Guangdong Provincial Engineering Technology Research Center for Healthy Breeding of Important Economic Fish, School of Life Sciences, Sun Yat-Sen University, Guangzhou 510275, China; huangmw@mail2.sysu.edu.cn (M.H.); chesterp2015@hotmail.com (C.P.); heicaonan1@163.com (X.W.); xiaoling459@126.com (L.X); wangdengdong@hotmail.com (D.W.); jiaxingchan@163.com (J.C.); lishuisheng219@126.com (S.L.); lsslhr@mail.sysu.edu.cn (H.L.); 3 Marine Fisheries Development Center of Guangdong Province, Huizhou 516081, China; zhhaifa812@Hotmail.com; 4 College of Ocean, Hainan University, Haikou 570228, China

**Keywords:** grouper, precocious sex reversal, hormone, efferent duct

## Abstract

In this study, we systematically investigated the process of sex reversal induced by 17-methyltestosterone (MT) feeding and MT-feeding withdrawal at the ovary differentiation stage in orange-spotted groupers, *Epinephelus coioides*. Gonadal histology showed that MT feeding induced a precocious sex reversal from immature ovaries to testes, bypassing the formation of an ovarian cavity, and MT-feeding withdrawal led to an ovarian fate. In both the MT feeding and MT-feeding withdrawal phases, cytochrome P450 family 11 subfamily B (*cyp11b*) gene expression and serum 11-KT levels were not significantly changed, suggesting that the MT-treated fish did not generate endogenous steroids, even though active spermatogenesis occurred. Finally, by tracing doublesex-expressing and Mab-3-related transcription factor 1 (dmrt1)-expressing cells and TUNEL (terminal deoxynucleotidyl transferase 2-deoxyuridine, 5-triphosphate nick end labeling) assays, we found that the efferent duct formed first, and then, the germ cells and somatic cells of the testicular tissue were generated around the efferent duct during MT-feeding-induced precocious sex reversal. Collectively, our findings provide insights into the molecular and cellular mechanisms underlying sex reversal induced by exogenous hormones during sex differentiation in the protogynous orange-spotted grouper.

## 1. Introduction

Groupers are hermaphroditic protogynous fish that develop ovaries at a young age and then undergo sex changes to become male later in life [1,2,3,4,5,6,7]. Artificial sex reversals are induced in many grouper species to overcome the shortages of mature males for breeding. Early studies in our lab have found that 17-methyltestosterone (MT) treatment can induce sex reversal in mature female groupers, but studies on androgen-induced sex reversal have focused on the genes related to the reproduction axis [8,9,10,11,12,13]. Permanent sex reversal from female to male was reported in response to exogenous androgen or aromatase inhibitors in groupers [14,15,16,17,18,19,20,21,22,23]. All of these artificial permanent sex reversals in groupers were performed when ovarian differentiation was complete, but whether MT treatment can induce a permanent sex reversal at the sex differentiation stage remains unexplored.

In gonochoristic fish, endogenous sex steroid hormones play a crucial role in sustaining phenotypic sex, and the most effective period to induce a sex change is during the gonadal sex differentiation stage [24]. In Nile tilapia (*Oreochromis niloticus*), exogenous androgen feeding can induce permanent sex reversal by suppressing *cyp19a1a* expression, which is essential for estrogen synthesis [25]. In hermaphroditic fish, the steroidogenic enzyme and serum sex steroid hormone levels vary during natural or induced sex changes [11,26,27]. Our previous study found that exogenous androgen treatment induced permanent sex reversals in orange-spotted groupers if endogenous androgens were produced [28]. Therefore, we proposed that endogenous androgens are important for maintaining male characteristics in fish. A permanent sex change may require a change in the expression patterns of the steroidogenic enzymes that generate endogenous androgens after a change from ovaries to testes.

For gonochoristic fish, a sex reversal in fish with undifferentiated bipotential gonads can be achieved easily by administering exogenous steroids or inducing adrenal insufficiency (AI). However, artificially induced sex reversal is difficult to achieve when gonad differentiation is complete [22,29,30]. In contrast, in hermaphroditic grouper species, MT treatment could not induce permanent sex reversal in immature females [31]. In a previous study, we found that the gonia-like cells in the germinal epithelium are the major source of male germ cells during sex reversal in 1.5-year-old groupers [28]. Gonia appear at 12 weeks after hatching, and gonad differentiation into ovaries happens at 17 weeks after hatching in the orange-spotted grouper [32]. The process of sex change at the sex differentiation stage needs more investigation.

In the present study, we analyzed the gonad histology, serum estrogen, and androgen levels, gene expression profiles and cellar changes during MT-feeding-induced sex reversal at the sex differentiation stage in orange-spotted groupers. Our results provide insights into the molecular and cellular mechanisms underlying impermanent sex reversal induced by exogenous hormones in this protogynous species.

## 2. Result

### 2.1. Gonadal Histology during MT Feeding

**Figure 1:**
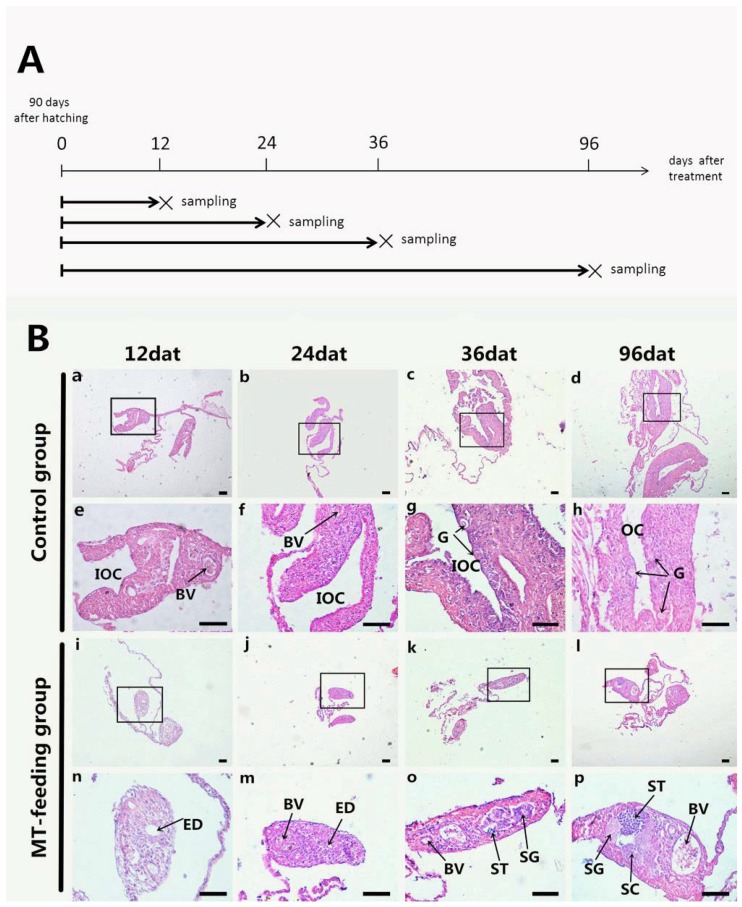
Gonad histology analysis during methyltestosterone (MT)-induced sex reversal. (**A**) Sampling time points. (**B**) Gonad histology. a–h: Gonad histology of control fish; i–p: Gonad histology of MT-treated fish; e–h: High magnification pictures of a–d, n–p: High magnification of the boxed areas in i–l. BV: blood vessel; IOC: initial ovarian cavity; OC: ovarian cavity; G: gonia; ED: efferent duct; SG: spermatogonia; SC: spermatocytes; ST: spermatids; Scale bars, 50 μm.

To induce sex reversal in groupers, we fed larvae at 90 days after hatching a diet containing MT at a concentration of 10 mg/kg diet. Sampling time points are illustrated in Figure 1A. The gonads from the control fish showed typical ovarian development, from the initial ovarian cavity (IOC) to a complete ovarian cavity (OC) with gonia (Figure 1B(a–h)). At 12 day after treatment (dat), the IOC of fish in the MT-feeding group disappeared with formation of the efferent duct (ED) (Figure 1B(i,n)). From 24 to 36 dat, a large number of spermatogonia (SG) appeared in the MT-fed group (Figure 1B(j,m,k,o)). At 96 dat, the gonads became testes and were full of SG, spermatocytes (SC), spermatids (ST) and ED tissue (Figure 1B(i,p)). These data indicate that MT feeding can effectively induce a male fate in groupers over a few weeks during the sex differentiation stage.

### 2.2. Gene Expression Profiles and Serum Steroid Hormone Levels during MT-Feeding-Induced Sex Reversal

**Figure 2:**
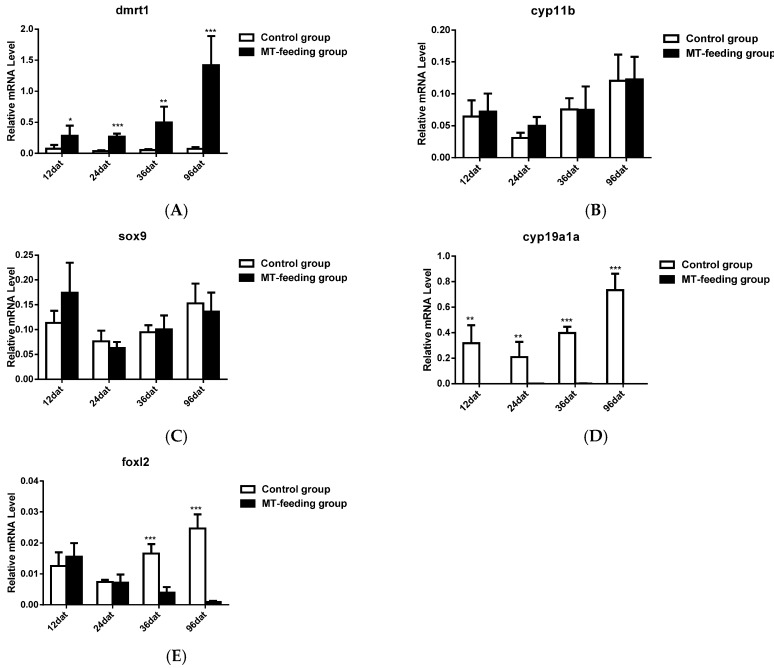
Expression profiles of key genes known to be involved in sex differentiation during MT-feeding induced sex reversal. (**A**–**C**), Gene expression of male-related genes *dmrt1* (**A**), *sox9* (**B**) and *cyp11b* (**C**) during MT-induced sex reversal (**D**,**E**), Gene expression of female-related genes *cyp19a1a* (**D**) and *foxl2* (**E**) during MT induced sex reversal. *β*-actin was used as the internal control. Data are expressed as the mean ± SD for three replicates obtained from five fish samples. *, ** and *** represent significant difference at *p* < 0.05, *p* < 0.01 and *p* < 0.001 between the control group and the MT-feeding group.

The expression level of key sex differentiation genes were analyzed during MT-feeding-induced sex reversal. The gene expression of *dmrt1* significantly increased from 12 to 96 dat (Figure 2A), and *cyp11b* and *sox9* expression were not significantly changed during this period (Figure 2B,C). On the other hand, the expression of *cyp19a1a* and *foxl2* significantly decreased during MT feeding (Figure 2D,E). These results indicate that the expression of female pathway genes (*cyp19a1a* and *foxl2*) was suppressed and that of male pathway genes (*cyp11b* and *sox9*) was not fully activated during MT-feeding at the sex differentiation stage.

**Figure 3:**
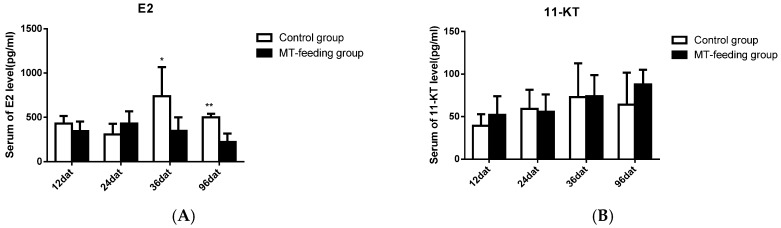
Serum E2 and 11-KT level during MT-induced sex reversal. (**A**) The E2 level in the control and MT-feeding group. (**B**) The 11-KT level in the control and MT-feeding group. Data are expressed as the mean ± SD for five fish. * and ** represent significant difference at *p* < 0.05 and *p* < 0.01 between the control group and MT-feeding group.

Compared with levels in control fish, serum E2 levels significantly decreased from 36 to 96 dat in the MT-fed group (Figure 3A), but serum 11-KT levels were not significantly changed during the same period (Figure 3B). These results indicate that MT-feeding decreases estrogen levels but it has no effect on androgen levels.

### 2.3. Dmrt1 Expression, TUNEL Staining, and Immunohistochemistry Analysis of Germ Cells during MT-Feeding-Induced Sex Reversal

**Figure 4:**
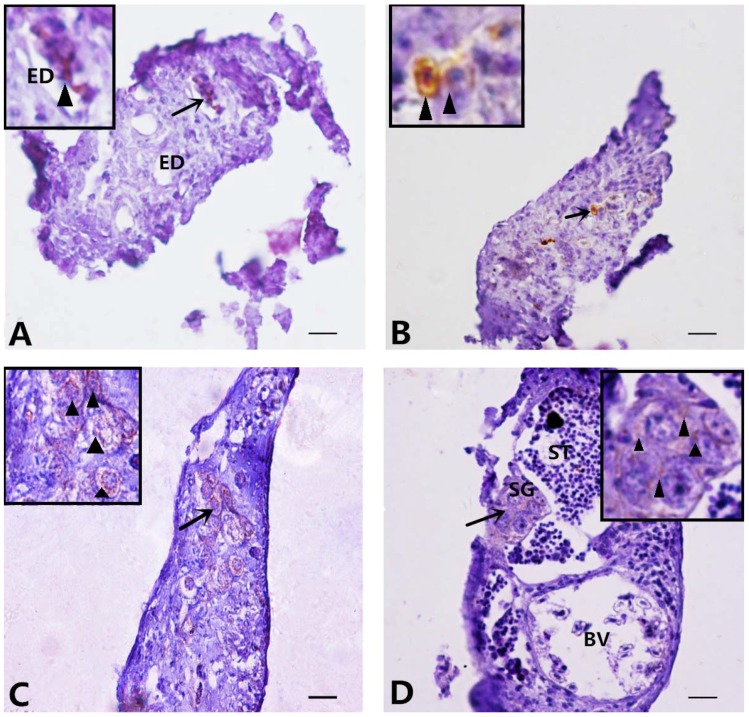
Tracing of Dmrt1-expressing cells during MT-feeding-induced sex reversal. The black arrows show the high magnification of the picture. Scale bars, 25 μm. Dmrt1 is detected in goniae cells around the efferent dust (black arrowheads) of MT-feeding fish at 12 dat (**A**). Dmrt1 is detected in a few of spermatogonia scattered in the gonads (black arrowheads) at 24 dat (**B**), and then they appear in gathered spermatogonia (black arrowheads) at 36 dat and 96 dat (**C**,**D**) of the MT-feeding fish. ED: efferent dust; BV: blood vessel; SG: spermatogonia; ST: spermatid.

To investigate where male germ cells originate during MT-feeding-induced masculinization, we examined Dmrt1 expression using immunohistochemistry (IHC). Dmrt1-positive signals were detected in cells around the ED and in sporadic cells at 12 and 24 dat in the MT-fed fish, respectively (Figure 4A,B). At 36 dat, Dmrt1-positive signals appeared in SG (Figure 4C). At 96 dat, positive signals were also observed in SG (Figure 4D).

**Figure 5:**
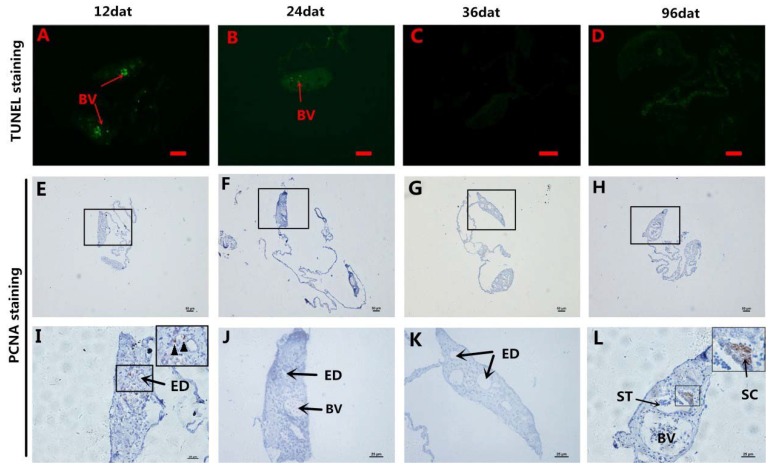
TdT-mediated dUTP nick end labeling (TUNEL) assay and Proliferating cell nuclear antigen (PCNA) staining. (**A**–**D**) TUNEL staining of gonad sections during MT-induced sex reversal. No TUNEL positive signals were detected in the 12 to 96 dat gonads tissues except in blood vessels (**A**–**D**). (**E**–**L**) The PCNA staining of gonad sections during MT-induced sex reversal. (**E**–**H**): High magnification of the boxed areas in (**I**–**L**). PCNA-positive signals were detected in somatic cells around the efferent dust (black arrowheads) in 12 dat gonad (**E**,**I**) and the SC in 96 dat gonad (**H**,**L**). BV: blood vessels; ED: efferent dust; SC: spermatocytes; ST: spermatids; Scale bars, 25 μm.

TUNEL (terminal deoxynucleotidyl transferase 2-deoxyuridine, 5-triphosphate nick end labeling) staining showed no positive signals in gonadal tissue except for a few cells in the blood vessels at 12 and 24 dat (Figure 5A–D). Proliferating cell nuclear antigen (PCNA) staining showed positive signals in cells around the ED at 12 dat (Figure 5E,F), but no positive signals were observed at 24 and 36 dat in gonads (Figure 5F,J,G,K). At 96 dat, PCNA-positive signals were observed in SC (Figure 5H,L). These results indicate that male germ cells probably originated from cells around the ED.

### 2.4. Gonadal Histology after MT-Feeding Withdrawal

**Figure 6:**
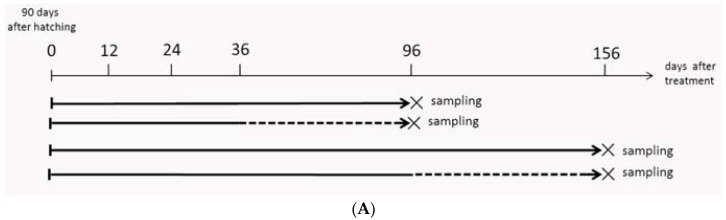

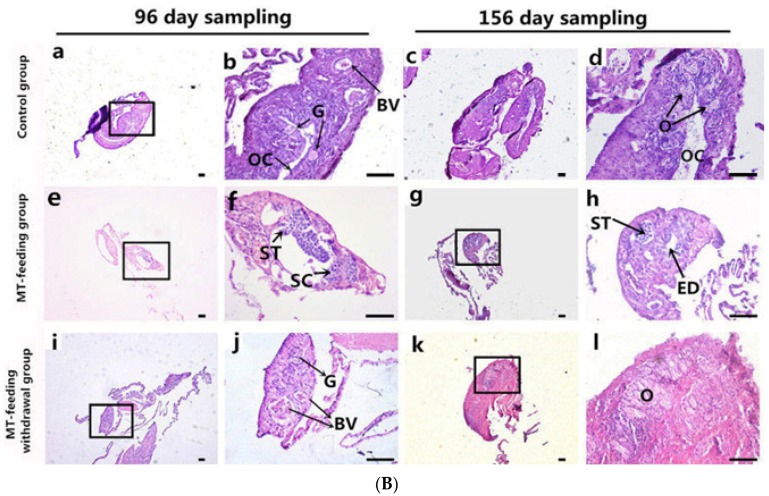
Gonad histology analysis after MT-feeding withdrawal. (**A**) Sampling time points. (**B**) Gonadal histology of the untreated control, MT-feeding and MT-feeding withdrawal groups. The panel b, d, f, h, j and l are high magnification pictures of a, c, e, g, i and k. G: gonia; OC: ovarian cavity; O: oogonium; ED: efferent dust; SG: spermatogonia; ST: spermatid; Scale bars, 25 μm.

To investigate whether the MT-feeding-induced male fate could be maintained, MT treatment was terminated at MT feeding for 36 or 96 days, and gonad samples were collected 60 days later (Figure 6A).

At both the 96 day and 156 day sampling points, the gonads of the control fish contained numerous oogonia, both groups had complete OCs (Figure 6B(a–d)), and the gonads of the MT-fed fish were full of SG, SC, and ST (Figure 6B(e–h)). However, in the 36 day MT-feeding-withdrawal group, their gonads were full of oogonia, but there were no OCs (Figure 6B(i,j)). In the 96 day MT-feeding-withdrawal group, the gonads contained a large number of oogonia, but OCs had not formed (Figure 6B(k,l)). These data suggest that the MT-feeding-induced male fate is not stable.

### 2.5. Gene Expression Profiles and Serum Steroid Hormone Levels after MT-Feeding Withdrawal

**Figure 7:**
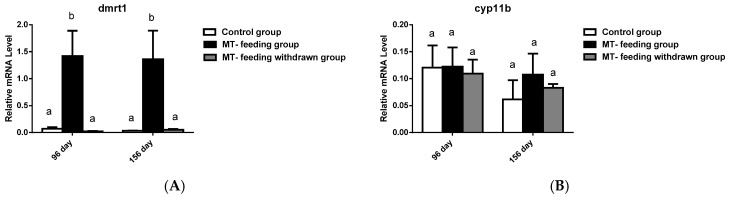

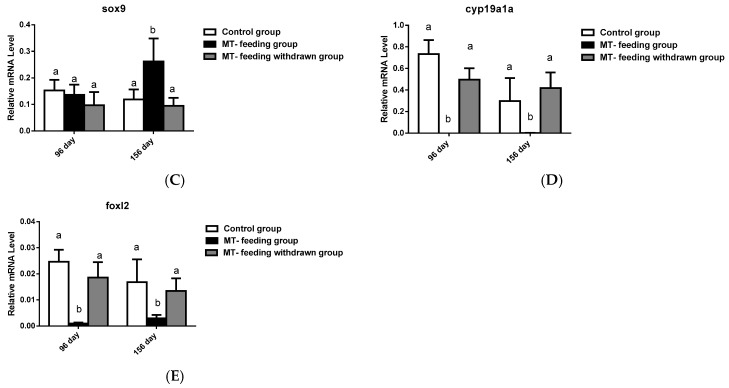
Expression profiles of key genes known to be involved in sex differentiation after MT-feeding withdrawal. (**A**–**C**) showed the expression of male-related genes *dmrt1*, *sox9*, and *cyp11b* at the 60th day after MT feeding withdrawal, respectively. (**D**,**E**) showed the expression of female-related genes *cyp19a1a* and *foxl2* at the 60th day after MT feeding withdrawal. *β*-actin was used as the internal control. Data are expressed as the mean ± SD for three replicates obtained from five different fish. Different letters indicate significant values at *p* < 0.05.

The expression of key genes involved in sex differentiation was further analyzed after MT-feeding withdrawal. The expression levels of the male and female pathway genes (*dmrt1*, *cyp11b*, *sox9*, *cyp19a1a* and *foxl2*) were not significantly different from expression in the control group at both 36-day and 96-day of MT-feeding withdrawal (Figure 7A–E). These results indicate that the MT-feeding-induced male fate cannot be maintained after MT-feeding withdrawal.

**Figure 8:**
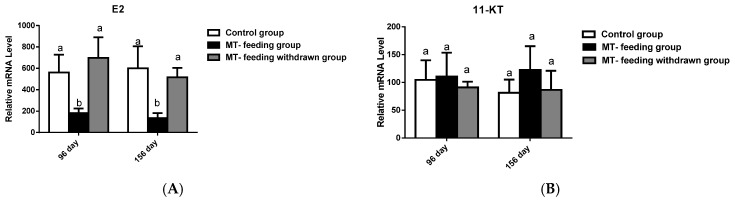
Serum E2 and 11-KT level after MT-feeding withdrawal. (**A**) The E2 level in the control, MT-feeding and MT-feeding withdrawn groups. (**B**) The 11-KT level in the control, MT-feeding, and MT-feeding withdrawn groups. Data are expressed as the mean ± SD for five fish. Different letters indicate significant values at *p* < 0.05.

We next examined serum E2 and 11-KT levels after MT-feeding withdrawal. E2 levels and 11-KT levels were not significantly different from those of the control group in both the 36-day and 96-day MT-feeding-withdrawal group (Figure 8A,B). These results also suggest that the MT-feeding-induced male fate is not stable.

## 3. Discussion

Our study shows that MT-feeding-induced sex reversal in 90-day-old orange-spotted groupers bypasses OC formation and that MT-feeding withdrawal leads to ovary development. These results indicate that MT-feeding-induced sex reversal during the sex differentiation stage is impermanent. A study on 120-day-old Malabar groupers (*Epinephelus malabaricus*) and 7-month-old juvenile female orange-spotted groupers also showed that MT treatment can induce a change of immature female fish into impermanent males [31,33]. On the other hand, MT has been reported to induce a permanent sex reversal from mature females to males in groupers [10,12,14,17]. These results suggest that whether the MT-treatment-induced sex reversal is stable is largely dependent on the gonadal stage in groupers.

The formation of an OC is an important step in fish developing toward a female fate. Interestingly, we found that MT-treatment abolished OC formation. OC formation was also prevented by eight weeks of MT feeding in 70-day-old red-spotted groupers, *Epinephelus akaara*, which is the age at which the OC formation stage occurs in red-spotted groupers [34]. Therefore, MT treatment can switch the female fate to the male fate by disrupting OC formation.

During natural or induced sex reversal at the adult age, *cyp19a1a* expression and serum E2 levels were decreased, whereas *cyp11b* expression and serum 11-KT levels were increased in groupers [7,26,27,35]. In this study, we found that the *cyp19a1a* expression and serum E2 levels significantly decreased during MT-feeding in 90-day-old orange-spotted groupers. The decreased estrogen level may be the key factor for inducing a female-to-male sex reversal. We also found that *cyp11b2* expression and serum 11-KT levels were not significantly changed. Similar results were reported for the MT treatment of immature Malabar groupers [31]. A recent study suggests that there are two types of steroid-producing cells, estrogen-producing cells, and androgen-producing cells in the gonads of the immature Malabar groupers [36], demonstrating that immature groupers have a chaotic endocrine system. MT, as an androgen, may directly stimulate spermatogenesis; however, large numbers of androgen-producing cells may not be produced during MT feeding, which may explain why MT treatment at this stage cannot induce permanent sex reversal.

In the MT-treated groupers, the OC was not formed and the ED developed in the gonads. Meanwhile, male germ cells and proliferating somatic cells appeared, surrounding the ED. These cells may be the major source of SG and pre-Sertoli cells. In Nile tilapia, it was reported that degenerated oocytes formed around the ED, and then SG, pre-Sertoli cells, and epithelial cells surrounding the ED appeared at the early stage of the induced female-to-male sex reversal [30]. A similar phenomenon has been found in mice [37]. Therefore, the formation of the ED is a key step for testis development, given that the germ cell and somatic cell (Sertoli cells, Leydig cells and epithelial cells) composition of the testicular tissue is first determined around the ED.

Our previous study found that gonia-like cells in the germinal epithelium might be the major germ cell source for developing testes during sex reversal in 1.5-year-old groupers [28]. In this study, we found that the Dmrt1-positive cells were first found among the gonia-like cells around the ED, and later, Dmrt1-positive SG were observed, suggesting that these gonia-like cells around the ED may be the source of male germ cells during MT-induced sex reversal in groupers at the sex differentiation stage. In mice, ovarian granulosa cells can transdifferentiate into testicular Sertoli cells, and Sertoli cells can transdifferentiate into granulosa cells during sex reversal [38,39]. In medaka, atretic follicles are the potential source of somatic cells in newly formed testicular tissue [40]. In this study, the gonads contained neither granulosa cells nor atretic follicles, but proliferating somatic cells were found surrounding the ED. These cells are a potential source of somatic cells in the newly developed testicular tissue, but whether these somatic cells are Sertoli cells, Leydig cells, or epithelial cells needs further investigation.

## 4. Materials and Methods

### 4.1. Animals

Ninety-day-old orange-spotted groupers (body mass, 2.18–3.22 g; body length, 4.4–5.8 cm) were obtained from the Guangdong Daya Bay Fishery Development Center (Huizhou, China) in April 2015. The reproductive season of groupers is from April to October in this bay. The orange-spotted groupers were at the ovarian stage of sex differentiation (ovarian cavity (OC) formation) based on gonadal histology. All animal experiments were conducted in accordance with the guidelines and approval of the respective Animal Research and Ethics Committees of Sun Yat-Sen University (ZSYQ201410, 1 January 2015).

### 4.2. MT-Feeding-Induced and MT-Feeding-Withdrawal-Induced Sex Reversal

We divided the fish into a control group and an MT-feeding group in two separate seawater tanks. Fish in the MT-feeding group (*n* = 120) were fed food mixed with MT (the MT was dissolved in 96% ethanol and added to the commercial fish feed at a concentration of 10 mg/kg diet), whereas the control fish (*n* = 60) were fed food that was mixed with 96% ethanol only. Five fish for each group were sacrificed, and gonadal tissues and serum samples were collected at 12, 24, 36, and 96 days after treatment (dat).

To analyze the effects of MT-feeding withdrawal on the sex fate, we removed 30 fish from the MT-feeding group at 36 and 96 dat. Two months after MT-feeding withdrawal, the gonadal tissues and serum samples were collected. The time table and the sampling program are shown in Figure 4A.

### 4.3. Gonadal Histology

Fish were sacrificed and their gonads were dissected, fixed in Bouin’s fluid, and then embedded in paraffin wax. Subsequently, they were applied to histological analysis through hematoxylin and eosin (H&E) staining. The size, sample number and the gonadal status of the sampled fish can be found in the Table 1.

### 4.4. Serum Estradiol-17β (E2) and 11-Ketotestosterone (11-KT) Assays

Blood were collected from the caudal vein and stored over crushed ice, the plasma was removed after centrifugation for 5 min at 10,000× *g*, and serum samples were stored at −20 °C until analysis. Serum E2 and 11-KT levels were measured using EIA(ELISA) kits (Cayman Chemical Co, Michigan, Ann Arbor, USA ), as described in a previous study [31].

### 4.5. RNA Isolation, Reverse Transcription and Quantitative Real-Time PCR

Total RNA was extracted from each sample and reverse transcribed using a Transcriptor First Strand cDNA Synthesis Kit (Roche, Basel, Switzerland) according to the manufacturer’s instructions. Real-time polymerase chain reaction (PCR) was performed on a Roche LightCycler 480 real time PCR system according to the manufacturer’s instructions. The real-time PCR conditions were as follows: denaturation at 94 °C for 1 min, followed by 40 cycles of 94 °C for 15 s, 55 °C for 15 s, and 72 °C for 60 s. Standard amplification curves for different genes were generated via serial dilutions of plasmid constructs. The concentration of the template in the samples was determined by relating the C_T_ value to the standard curve. The *β*-actin gene was used as the internal control. Primers used in this study are described in previous research [28] and are listed in Table 2.

### 4.6. Immunohistochemistry and TUNEL Staining

IHC analyses were performed as described previously [28]. Antibodies against Dmrt1 and proliferating cell nuclear antigen (PCNA) (Cambridge, UK) were diluted at a ratio of 1:100 and 1:1500. Apoptosis in gonads was examined by a TUNEL assay with an In Situ Cell Death Detection kit, POD (Roche Molecular Biochemicals) following the manufacturer’s instructions. The sections were counterstained with hematoxylin after TUNEL staining. Sections processed without terminal deoxynucleotidyl transferase were used as a negative control. Photographs of the samples were taken under a Niko light microscope (Minato-ku, Tokyo, Japan).

### 4.7. Statistical Analysis

Quantitative data were expressed as the mean ± SD. Significant differences were analyzed using a two-way analysis of variance (ANOVA) (GraphPad 5.0). A probability level of less than 0.05 indicates a significant difference.

## 5. Conclusions

In conclusion, our study provides several insights into the process of MT-induced sex reversal during sex differentiation in groupers. First, MT-feeding-induced sex reversal is impermanent at this stage, because the endogenous androgen pathway is not fully activated. Second, disrupted OC formation and the appearance of the ED are early signs of female-to-male sex reversal. Third, male germ cells and newly developed testicular somatic cells probably originated from the gonia-like cells and proliferating somatic cells surrounding the ED, respectively.

## Figures and Tables

**Table 1:** The size and gonadal status of *Epinephelus coioides* during experimental period.

Duration of Administration	Sample No	Body Weight ^e^ (g)	Total Length ^e^ (cm)	Gonadal Status			
				Ovary ^a^	Ovary ^b^	Testis ^c^	Testis ^d^
Initial control at 90 day after hatching	8	4.02 ± 0.30	6.69 ± 0.16	5	0	0	0
Control group							
12 dat	5	5.32 ± 0.48	6.78 ± 0.16	5	0	0	0
24 dat	5	6.76 ± 0.52	7.84 ± 0.19	5	0	0	0
36 dat	5	13.9 ± 0.65	9.64 ± 0.25	3	2	0	0
96 dat	5	22.72 ± 1.74	11.84 ± 0.25	0	5	0	0
156 dat	5	55.72 ± 2.80	16.06 ± 0.36	0	5	0	0
MT treatment group							
12 dat	5	6.77 ± 1.52	7.22 ± 0.54	0	0	5	0
24 dat	5	7.16 ± 1.00	7.70 ± 0.45	0	0	5	0
36 dat	5	11.77 ± 0.79	9.22 ± 0.20	0	0	1	4
96 dat	5	20.56 ± 2.05	10.48 ± 0.47	0	0	0	5
156dat	5	50.36 ± 1.99	15.84 ± 0.72	0	0	0	5
MT termination group							
96 dat	5	21.28 ± 0.92	10.30 ± 0.72	0	5	0	0
156 dat	5	58.18 ± 1.50	16.1 ±0.65	0	0	0	5

^a^ ovary: contain initial ovarian cavity. ^b^ ovary: contain ovarian cavity. ^c^ Testis: contain efferent duct and there are not many male germ cells. ^d^ Testis: contain many male germ cells. ^e^ Mean ± SD.

**Table 2:** Nucleotide sequences of the primers used in this study.

Primers	Primers Sequence (from 5′ to 3′)
Primers for real-time polymerase chain reaction (PCR)	
dmrt1-F	GCTGGAGTAGACTGCTTGTTT
dmrt1-R	CGACTGTGCGTCAGTATGAGC
cyp11b-F	TGTTGCCGTCTGACATCG
cyp11b-R	TCGCCACTCCTCACCGTTC
sox9-F	GCAATGCAGGCTCAGAATAG
sox9-R	GGTATCAAGGCAGTACCCAG
cyp19a1a-F	GGAGACATTGTGAGAGTCTGGATC
cyp19a1a-R	TGACAGGTACATCCAGGAAGAGTC
foxl2-F	CCACCGTACTCCTATGTCGC
foxl2-R	GTCTGATACTGTTCTGCCAAC
*β*-actin-F	ACCATCGGCAATGAGAGGTT
*β*-actin-R	ACATCTGCTGGAAGGTGGAC
